# High coverage COVID-19 mRNA vaccination rapidly controls SARS-CoV-2 transmission in Long-Term Care Facilities

**DOI:** 10.21203/rs.3.rs-355257/v1

**Published:** 2021-04-12

**Authors:** Pablo M De Salazar, Nicholas Link, Karuna Lamarca, Mauricio Santillana

**Affiliations:** 1 Center for Communicable Disease Dynamics, Department of Epidemiology, Harvard TH Chan School of Public Health, Boston, United States; 2 Department of Biostatistics, Harvard TH Chan School of Public Health, Boston, MA; 3 Machine Intelligence Lab, Boston Children’s Hospital, Boston, United States; 4 Computational Health Informatics Program, Boston Children’s Hospital, Boston, United States; 5 Department of Pediatrics, Harvard Medical School, Harvard University, Boston, United States; 6 Home Hospitalization Unit, Department of Infectious Diseases, Dos de Maig Hospital, Universitat Autònoma de Barcelona, Barcelona, Spain

**Keywords:** COVID-19, vaccination, long-term-care facilities, time series analysis, vaccine effectiveness

## Abstract

Residents of Long-Term Care Facilities (LTCFs) represent a major share of COVID-19 deaths worldwide. Information on vaccine effectiveness in these settings is essential to improve mitigation strategies, but evidence remains limited. To evaluate the early effect of the administration of BNT162b2 mRNA vaccines in LTCFs, we monitored subsequent SARS-CoV-2 documented infections and deaths in Catalonia, a region of Spain, and compared them to counterfactual model predictions from February 6th to March 28th, 2021, the subsequent time period after which 70% of residents were fully vaccinated. We calculated the reduction in SARS-CoV-2 documented infections and deaths as well as the detected county-level transmission. We estimated that once more than 70% of the LTCFs population were fully vaccinated, 74% (58%−81%, 90% CI) of COVID-19 deaths and 75% (36%−86%) of all documented infections were prevented. Further, detectable transmission was reduced up to 90% (76–93% 90%CI). Our findings provide evidence that high-coverage vaccination is the most effective intervention to prevent SARS-CoV-2 transmission and death. Widespread vaccination could be a feasible avenue to control the COVID-19 pandemic.

## Introduction

Widespread vaccination has the potential to significantly reduce SARS_CoV-2 infections and deaths, and subsequently improve social and economic conditions [1]. Available mRNA COVID-19 vaccines have been approved due to their capacity to reduce symptomatic disease, hospitalizations, and deaths in clinical trials [2,3], but evidence of their real-world effectiveness remains limited [4,5].

Among all populations, residents of Long-Term Care Facilities (LTCFs) represent a major share of COVID-19 deaths, with a 7-fold higher incidence of death compared to the general population in the US [6,7]. As a consequence, they have been prioritized for vaccinations in most settings. While observational data post-vaccination are scarce to date, particularly regarding LTCFs [8], early assessments on whether clinical trial results are good indicators of vaccine effectiveness in LTCFs would help refine control strategies [1].

In this study, we aimed to quantify the early effect of the administration of the BNT162b2 mRNA COVID-19 vaccine on reducing the risk of SARS-CoV-2 transmission and COVID-19 death in LTCFs in Catalonia (Spain).

## Methods

Documented COVID-19 infections, identified with a molecular test (PCR or antigen) independently of symptoms, were assumed to capture most infections. Documented deaths attributable to COVID-19 included those with laboratory confirmation and those only meeting clinical and epidemiologic criteria. Detected county-level transmission was used as an indicator of transmission and defined as at least one documented infection in any facility within a county per unit of time. LTCFs residents were defined as those living in a LTCFs and older than 64y. The general population, or “community”, was defined as all people in a county not living in LTCFs. All vaccinated individuals received the two doses of the BNT162b2 mRNA vaccine. For further details see Supplementary Information Section S1.

We generated multiple time series of daily confirmed infections, deaths, and vaccinations in LTCFs, aggregated by healthcare area level (n=9), and in the broader Catalonia region. Similarly, we collected daily confirmed infections in the general population, at the healthcare-area level and regional level. For each of these time series, we took a moving weekly average around each day to smooth some of the daily variation of reporting. Further, we generated a time series of the detected county-level transmission (n= 41, “comarques”) by week. The pre-vaccination period was from July 6 to December 27 2020, when vaccination in LTCFs began. We evaluated the impact of vaccines during an evaluation time period from February 6 to March 28, 2021, the subsequent time period after which 70% of residents were vaccinated with two doses. The 70% threshold was chosen to represent the estimated herd immunity - the estimated level of immunity in a population that prevents uncontrolled spread of infections [9]. As a sensitivity test, we evaluated the effect of partial vaccination in a longer period starting on January 14, when 70% of residents had received the first dose of the vaccine.

We built regression models to predict the number of infections and deaths using community infections as inputs. These models were calibrated during the pre-vaccination period and then used to generate predictions in the absence of vaccines during the evaluation period. We compared the models’ counterfactual predictions with observations; discrepancies were used to quantify the effects of the vaccine. Further, for each county with at least one pre-vaccination week with a transmission event and at least one week without one (n=36), we built logistic regressions to estimate the probability that a documented transmission would be observed, using community infections as input. These models were trained on the pre-vaccination period and then used to predict transmission events in the evaluation period; the aggregated county-level predicted probabilities and the aggregated observed transmissions were compared to quantify vaccine effectiveness. See Supplementary Information Section S1 for further details on the models.

## Results

We quantified the effect of administering the BNT162b2 vaccine (a) on reducing deaths and documented infections among residents older than 64y, and (b) on reducing detected transmission caused by SARS-CoV-2 in LTCFs in Catalonia.

**Figure 1.**
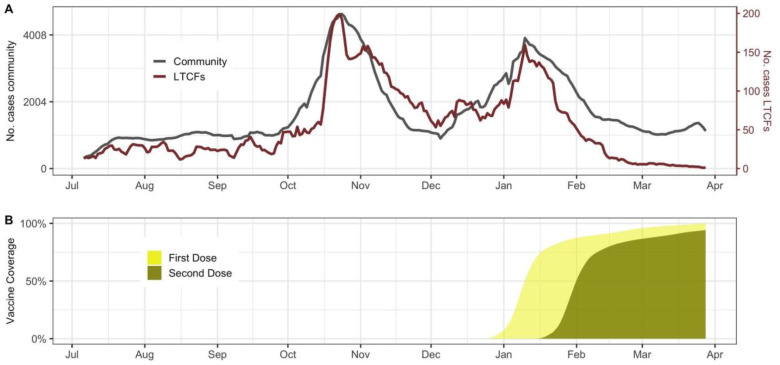
A) Comparison of the total community (grey) and LTCFs documented infections (red) trajectories in Catalonia, Spain. B) First and second dose vaccine coverage among LTCFs residents

Figure 1 A shows the temporal evolution of infections documented in the community and in LTCFs between July 6, 2020 - March 28 2021. For context, the cumulative vaccination coverage among all LTCFs residents is shown in panel B. Vaccination was deployed among residents and healthcare workers at similar times across LTCFs facilities in the region, beginning December 27 and reaching more than 95% of 2-dose coverage within 2 months.

**Figure 2:**
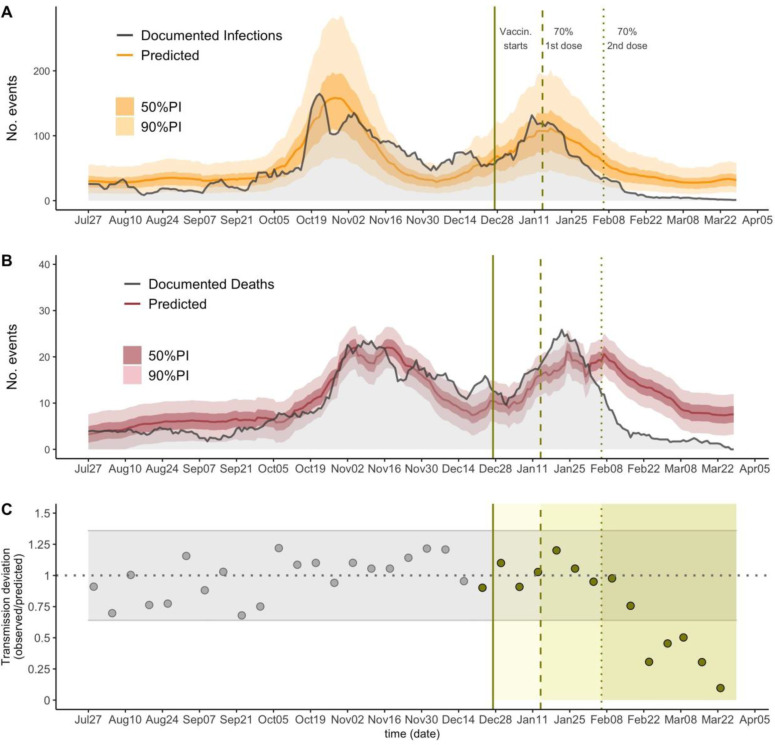
The predictions for infections (A) and deaths (B) across all of Catalonia. The solid lines show the model predictions from training July 6, 2020 through December 27, 2020, the darker shaded background shows the 50% prediction intervals (PI) and the lighter background shows the 90% PI. Vertical lines show key analysis time points: when vaccination started (solid), when 70% of residents received the first dose and when 70% of residents received the second dose. (C) The ratio between observed and predicted transmission at county level in Catalonia, represented by point estimates, grey for the training period and green for the prediction period; grey horizontal ribbon represents the 90% confidence interval. Solid green areas represent the prediction periods after vaccination starts.

Figure 2 A and B show predictions and observations of documented infections and deaths in all of Catalonia over time. We estimated that between February 6-March 28 2021, vaccines prevented 75% of documented infections (36% - 86%, 90% CI), and 74% of deaths (58% - 81%, 90% CI). As well, our analysis shows that two weeks after 70% of residents were fully vaccinated, detected transmission was significantly reduced by 69% (24–80% 90%CI), 54% (0–70%), 50% (0–68%), 69% (25–80%), and 90% (76–93% 90%CI) for each subsequent epidemiological week (Figure 2C).

## Discussion

In this study we show that high vaccination coverage (over 70%) prevented around 3 out of 4 expected COVID-19 deaths in subsequent weeks, which is consistent with the vaccine effect estimated in clinical trials [2] and observational studies [4,8].

Furthermore, LTCFs represent enclosed populations, where transmission is caused both by external introduction of the virus, mostly by the staff [10], and internal transmission. Observational findings from facilities with high infection-ascertainment, such as those studied here, may provide valuable information of what may be expected to happen in more general settings and populations. Given this, we conclude that beyond the specific risk factors of the population in LTCFs, such as age or comorbidities, our findings suggest that high-coverage vaccination can rapidly control SARS-CoV-2 transmission in an enclosed population. Particularly, our analysis suggests that transmission is reduced 3–10-fold one month after vaccination has reached 70% coverage.

Our methodological assumptions may lead to underestimation of the true vaccine effect. First, our definition of transmission events at the county-level does not capture the facility-level transmission; thus, our results may fail to capture more dramatic facility-level reductions in transmission. Second, the counterfactual estimates of deaths produced by our models may be underestimations of mortality during the evaluation period. Indeed, the documented deaths were generally higher than those estimated by our models in December and January (Figure 2B). It is plausible that factors not considered in the model, such as seasonality [11] and/or the spread of more lethal variants [12] may have increased COVID-19 mortality in recent times.

In addition, we acknowledge that our investigation has limitations. We assume that the rigorous screening standards in LTCFs in Catalonia led to infection ascertainment close to 100% and also that the ascertainment of community infections does not change dramatically over time. While this may appear unrealistic, we suspect it is not far from the truth, as public health authorities in Catalonia substantially increased the infection screening efforts in LTCFs before the vaccination campaign [13].

Further, regression models were not designed for accurate infection (or deaths) forecasting and as such, they may not fully capture the epidemiological dynamics. However, our efforts were focused on proper inference of the expected epidemic trajectory in the absence of vaccination. This goal is achieved as our models appear to reasonably capture the overall dynamics during the pre-vaccination time periods, even at high spatial granularity (Supplementary Information Figure S1–S2).

Finally, there could be unmeasured confounders, such as behavior or policy changes, not captured by our models, that may have changed the dynamics of transmission between the community and LTCFs over time --this may be the case in the health area Alt Pirineu i Aran (Supplementary Information Figure S1–S2). In most studied regions, however, we suspect this was not the case.

In spite of these limitations, our analyses provide evidence that vaccination may be the most effective intervention in controlling SARS-CoV-2 spread and subsequent risk of death available to date. If our findings continue to be confirmed by future studies, widespread vaccination could be shown to be a feasible avenue to control the COVID-19 pandemic.

## Supplementary Material

1

## Data Availability

All data was obtained from a publicly available repository https://www.dadescovid.cat.
