# Supplementary material for: High coverage COVID-19 mRNA vaccination rapidly controls SARS-CoV-2 transmission in Long-Term Care Facilities
Source: Res Sq. 2021 Apr 12:rs.3.rs-355257. Preprint. [Version 1] doi: 10.21203/rs.3.rs-355257/v1 (PMC8057244; doi:10.21203/rs.3.rs-355257/v1)
Supplement: 1 [file NIHPPrs355257v1-supplement-1.pdf]

Supplementary Information for: *"High coverage COVID-19 mRNA vaccination rapidly controls SARS-CoV-2 transmission in Long-Term Care Facilities"*

Section S1. *Supplementary Methods*

Details on ascertainment standards:

Starting in July 2020, LTCFs in Catalonia implemented rigorous COVID-19 surveillance that did not solely rely on detection of symptomatic cases [1]. All contacts among staff and residents are screened using molecular test (PCR or antigen test) immediately upon confirmation of an index infection in a facility; further, all staff and residents are regularly screened independently of whether the individuals show symptoms or not; public health guidelines require to screen staff every 2-4 weeks depending on the population size where the LTCFs is located. Thus, the amount of documented infection relies under all infections but over symptomatic infections (which have been estimated to be around ~60% in LTCFs/skilled nursing homes) [2,3]. For our analysis using this dataset, we assume that the number of documented infections and COVID019 deaths closely approximates the true number of infections and deaths. For example, in a facility where individuals are screened every 2 weeks [4] on top of having symptomatic surveillance and outbreak investigation standards, reporting no documented infections --when in reality there is transmission-- would require the full outbreak to remain undetected (i.e all infections occurring within the same outbreak remain asymptomatic), the outbreak to die off within days or weeks without isolation of exposed individuals, and the infections to show no detectable viral RNA in <14 days after infection (the median duration of detectable SARS-CoV-2 in unvaccinated individuals is estimated to be ~20 days [5]) . Similarly we assume that the event of not detecting any infection in any of the facilities of a county for a whole week closely approximates the true absence of transmission in the county. Deviations from these assumptions are discussed in the main text.

Estimating Infections and Deaths Averted By Vaccinations

To estimate the total infections and deaths averted, we aggregate data across all of Catalonia. While it might make sense to use point estimates of infections and deaths averted from individual counties, the more granular model fits are not as good and there is not a clear way to combine the uncertainty from these multiple estimates given their temporal and spatial correlation. We trained a negative binomial model (model 1, eq. 1) to predict the nursing home infections, N_d, for day *d* using community infections that day and one week prior, C_d and C_{d-7} respectively. N_d and C_d are moving weekly averages of documented infections.

$$log(E[N_d]) = B_0 + B_1 log(1 + C_d) + B_2 log(1 + C_{d-7}) + log(S_d) \quad \text{(eq. 1)}$$

where $S_d = 1 - Im_d$ and Im_d is the proportion of the population that is immune:

$$Im_0 = 0.1$$

$$Im_d = max(0, Im_{d-1}(1 - 0.18/365) - D_{d-1}/n + N_d/n)$$

We include an offset term $log(S_d)$, where S_d is the proportion of the population that is susceptible, to account for the expected decrease in infections due to susceptible depletion; including $log(S_d)$ amounts to multiplying predictions by the susceptible proportion. $D_d$is the weekly average of deaths on day *d*, the $(1 - 0.18/365)$ term accounts for a 18% turnover rate in nursing homes in a usual year [6], and n is the estimated nursing home population (n=57,922). Since the exact population size in LTCFs was not available at all considered spatial resolutions, we approximated this quantity by the maximum number of vaccinated people once vaccination was reported complete (n=57,922).

For deaths, we use a similar model (model 2, eq. 2), though we use a zero-intercept linear regression of community infections because the relationship between number of infections and number of deaths is likely linear (because a specific proportion of nursing home infections weeks prior will be expected to die).

$$E[D_d] = B_1 C_d + B_2 C_{(d-7)} + B_3 C_{(d-14)} + B_4 C_{(d-21)} \quad \text{(eq. 2)}$$

The model was trained on data from a baseline period, July 6, 2020 to December 27, 2020, and then applied to data from an evaluation period, December 28 to March 28 (on December 26, nursing homes began administering vaccines). We generated model fits and prediction intervals in the evaluation period. The prediction intervals for deaths (eq. 2) were generated by using R's prediction function and the prediction intervals for infections (eq. 1) were generated using a parametric bootstrap procedure. To estimate the number of infections or deaths averted during a target period, we summed the daily model fits and bounds of the prediction intervals. This yields wider intervals than is likely true but given the temporal correlation of the predictions it would not be sensible to combine the variances of each prediction as if they were independent. We selected two target periods to compute averted infections and deaths based on the dates when 70% of the nursing homes residents received the first vaccine dose and when the same proportion received the second dose January 14 and February 6. This allowed us to adjust for changes over time of the vaccines' effect given the delay between vaccination and efficacious immunization, as well as to account for vaccine coverage close to herd immunity estimates (assumed >70%, see main text). As a supplementary analysis (see supplementary analysis, section S2), we estimated the number of deaths per LTCFs infection, which approximates the mortality rate, before and after vaccination.

Predicting change in probability of detected transmission in facilities

We analyzed the changes in detected transmission at the county level. We define a detected transmission occurrence, O_{iw}, as at least one COVID infection among nursing homes residents in county *i* for week *w*. We predict the probability of a transmission occurrence, $\pi_{iw} = P(O_{iw})$, using the logistic regression model (model 3, eq.3).

$$logit(\pi_{iw}) = B_0 + B_1 C_{iw} + B_2 C_{i(w-1)} + log(S_{iw}) \quad \text{(eq. 3)}$$

Counties without any detected transmission during the pre-vaccination period (1 county) or without any weeks with no transmission (4 counties) were excluded from this analysis because of the inability to fit a logistic regression model with only one outcome class. To avoid overfitting,

we calculated leave-one-out predictions during the pre-vaccination period and out-of-sample predictions for the vaccination period using a model trained in the pre-vaccination period. Then we computed the ratio of observed to predicted transmission events (denoted as $TD_w = Transmission - Deviation\ (week\ w)$) as an approximation of the vaccine effectiveness in fully preventing transmission.

$TD_w = \frac{\sum_i o_{iw}}{\sum_i \pi_{iw}}$ (eq. 4)

To generate confidence intervals around our predictions, we calculated the sample standard deviation (σ_{TD}) of the pre-vaccination predictions (around a mean ≈1) and use the normal distribution confidence intervals. We believe this is reasonable because we are summing 36 (41 - 5 excluded counties) individual (non-normal) distributions, and by the central limit theorem this should be approximately normal. To estimate the proportion of documented transmission averted with 90% confidence intervals, we calculated $1 - TD_w(1 - 1.645\sigma_{TD} - TD_w, 1 + 1.645\sigma_{TD} - TD_w)$.

All analysis was conducted using R 4.0.3 (https://www.R-project.org/).

Section S2. *Predicting change in epidemic size across healthcare areas.*

Due to the high zero-inflation of documented infections LTCFs at the county level and the difficulty this provides for statistical modeling and inference, we predicted the epidemic size at a higher aggregate level, named “regió sanitaria” (herein healthcare area, n=9). For each area, we predicted LTCFs infections using model 1 and LTCFs deaths using model 2 (see methods). We followed the same procedures as described in the methods to obtain the prediction intervals.

Consistent with the main analysis, healthcare area predictions showed lower-than-expected documented infections and deaths at a more granular level for both target periods of analysis, as seen in eFigure 1 A and B respectively. In 7 out of the 9 health areas the documented infections during the analysis period were consistently lower than the expected infections, and in the two areas where observed infections were higher (Barcelona Ciutat and Metropolitana Nord, among the most heavily populated) they became lower than expected in the final two weeks. Also, infections in Alt Pirineu i Aran become lower than expected around 1-2 weeks earlier than vaccine interventions and remain low for the target time periods, which likely reflect strong specific lockdown measures implemented there. Further, observed deaths were higher during the early analysis period (~January) in 5 out of 9 healthcare areas (Barcelona ciutat, Camp de Tarragona, Catalunya Central, Girona, Metropolitana Sud), and later became significantly lower, consistent with the main analisis. Factors not considered in the model such as variation of mortality rates due to seasonality or spread of variants with higher lethality might have biased our estimates; in this case, the true number of prevented deaths and infections would be bigger than those estimated by our model. eTable 1 and 2 summarize the number of documented infections and deaths averted by each healthcare area for the two target periods described in the methods. Note that because we used a linear model to predict deaths, some of the predictions are negative, which we truncated at zero. Due to the granularity of the area-level and the greater uncertainty around both models’ predictions, many of the infections- and

deaths-averted confidence intervals contain negative values, which would indicate an increase in either outcome. The locations with a greater population (Barcelona Ciutat and Metropolitana Nord) tend to have smaller confidence intervals because their greater population size yields more stable outcome values. While the area-level confidence intervals are wide, when we look at Catalonia as a whole (as in our main analysis) we see a more certain effect of vaccines.

Figure S1. The epidemic size predictions for documented infections by healthcare area

A

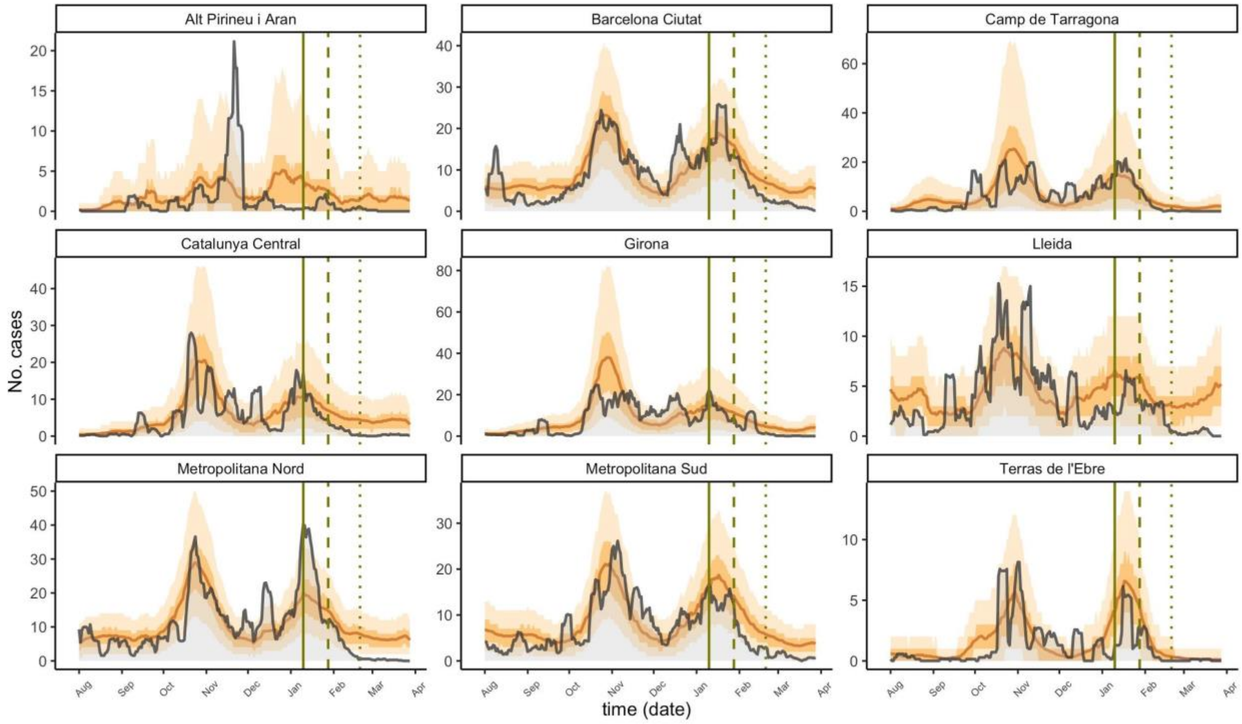

B

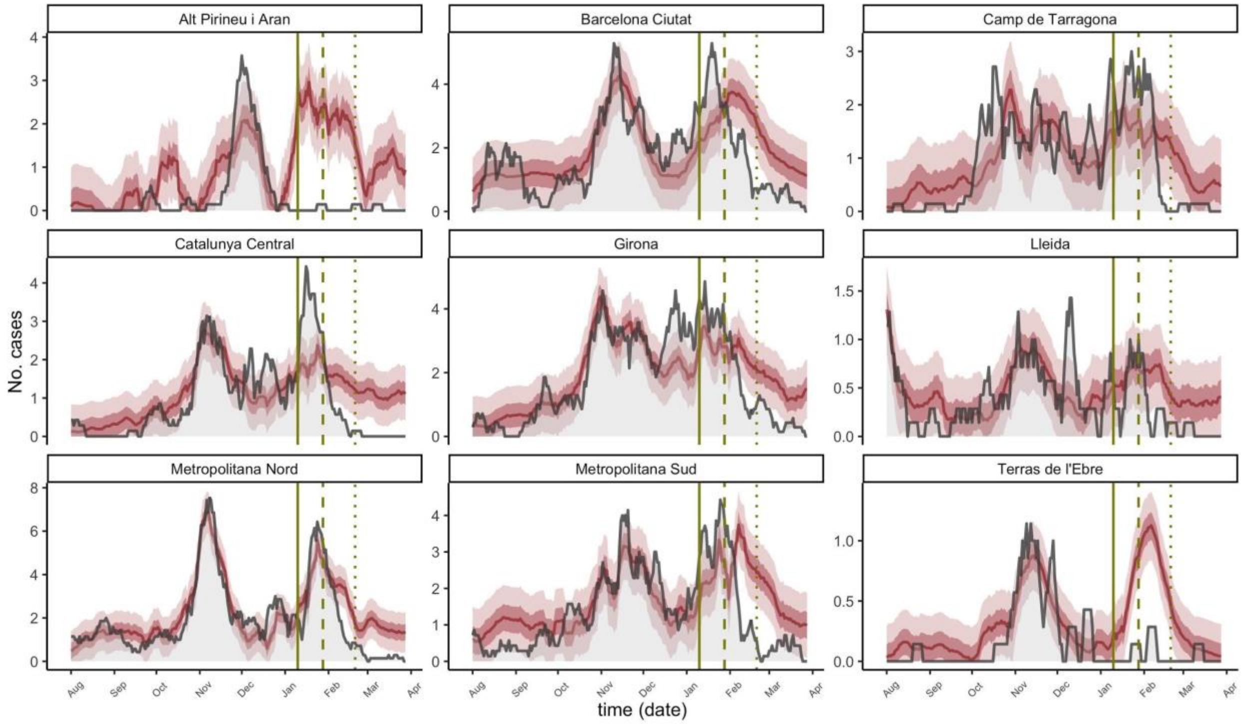

Figure S1. The grey lines are the observed documented infections (A) and deaths (B) and the yellow and red lines are the predicted infections, with ribbons for the IQR and 90% PI. Vertical lines show key analysis time points: when vaccination started (solid), when 70% of residents

received the first dose and when 70% of residents received the second dose. Negative predictions are truncated and plotted as 0 for consistency.

**Table S1 - Number of Documented Infections Averted**		
	**Since 70% first-dose vaccination - Jan 14, 2021 (90% CI)**	**Since 70% second-dose vaccination - Feb 6, 2021 (90% CI)**
All of Catalonia<br>*% averted (90% CI)*	1659 (0, 4817)<br>*42% (0%, 68%)*	1371 (157, 2867)<br>*75% (36%, 86%)*
Alt Pirineu i Aran	109 (0, 469)	72 (0, 283)
Barcelona Ciutat	174 (0, 753)	191 (0, 491)
Camp de Tarragona	49 (0, 685)	87 (0, 304)
Catalunya Central	255 (0, 836)	195 (0, 527)
Girona	190 (0, 843)	117 (0, 429)
Lleida	167 (0, 521)	134 (0, 354)
Metropolitana Nord	256 (0, 854)	311 (61, 644)
Metropolitana Sud	280 (0, 798)	182 (0, 437)
Terres de l’Ebre	50 (0, 243)	11 (0, 69)

Table S1: Number of predicted averted cases in all of Catalon and in each healthcare area. All values are cumulative estimates of cases averted between the starting dates, Jan 14, 2021 and Feb 6, 2021, and March 28, 2021. The 90% confidence intervals are the sums of the bounds of the daily 90% prediction intervals, so they are likely wider than in reality. Negative confidence interval values (indicating higher-than-expected cases) are truncated at 0.

**Table S2 - Number of Deaths Averted**		
	**Since 70% first-dose vaccination - Jan 14, 2021 (90% CI)**	**Since 70% second-dose vaccination - Feb 6, 2021 (90% CI)**
All of Catalonia<br>*% averted (90% CI)*	382 (55, 709)<br>*38% (8%, 53%)*	445 (220, 669)<br>*74% (58%, 81%)*
Alt Pirineu i Aran	117 (49, 185)	64 (17, 110)
Barcelona Ciutat	56 (0, 135)	67 (13, 121)

Camp de Tarragona	12 (0, 77)	31 (-14, 75)
Catalunya Central	37 (0, 89)	56 (19, 92)
Girona	44 (0, 112)	58 (11, 105)
Lleida	20 (0, 51)	18 (-4, 39)
Metropolitana Nord	60 (0, 128)	71 (24, 119)
Metropolitana Sud	54 (0, 121)	74 (28, 120)
Terres de l'Ebre	30 (10, 50)	15 (1, 29)

Table S2: Similar to TableS1, but for deaths averted.

Section S3. *Estimating changes in the fatality rates*

As sensitivity, we predicted the number of LTCFs deaths from LTCFs documented infections using the model 4 (eq. 5) in Catalonia.

$D_{rd} = B_1 N_{r(d-7)} + B_2 N_{r(d-14)} + B_3 N_{r(d-21)} + \epsilon_d$ (eq. 5)

This is a proxy for the fatality rate per infection. Interestingly, the fatality rates seem to increase in January (Figure S2) after vaccines are first delivered and decrease after later on, which could explain the excess of deaths observed in the main analysis compared to those predicted from community infections.

Figure S3. Predictions of the number of LTCFs deaths from LTCFs documented infections using the model 4 (eq. 5)

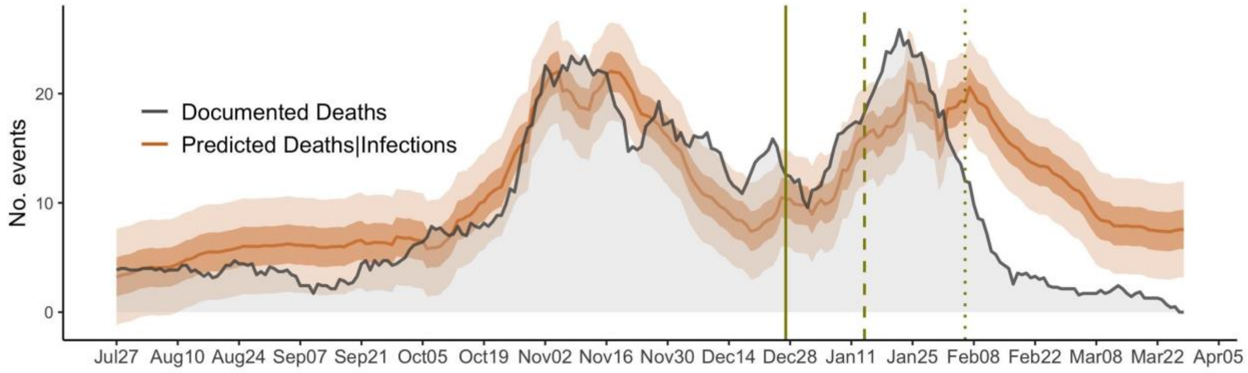

Figure S3: The grey lines are the documented deaths, and the brown lines are the predicted deaths, with ribbons for the 50%PI and 90% PI. Vertical lines show key analysis time points: when vaccination started (solid), when 70% of residents received the first dose and when 70% of residents received the second dose.

Section S4. *Percent Errors for Documented Infections and Death Models*

As sensitivity we computed the percent error over time for documented infections (model 1, eq 1) and deaths (model 2, eq 2). As shown in Figure S3 the trend consistently increased toward negative values after vaccination started. For both events, the deviation predictions vs observations during the target time period Feb 6- March 28 becomes close to -100%.

Figure S4

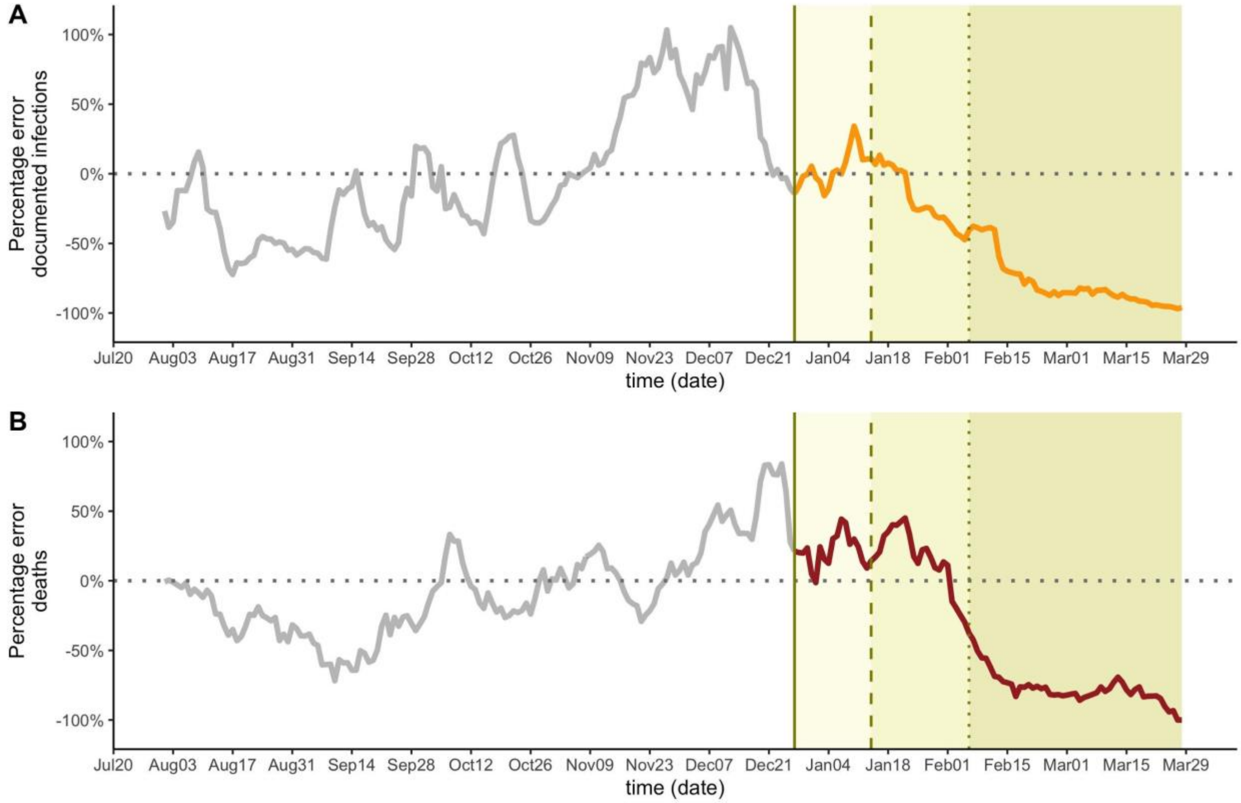

Figure S4. Solid lines represent daily percentage error for documented infections (A) and death model (B). Grey color is used for the training time period pre vaccination and orange and red once vaccination started. Vertical lines show key analysis time points: when vaccination started (solid), when 70% of residents received the first dose and when 70% of residents received the second dose.
